# Analysis of the Subgingival Microbiota in Implant-Supported Full-Arch Rehabilitations

**DOI:** 10.3390/dj8030104

**Published:** 2020-09-05

**Authors:** Maria Menini, Francesca Delucchi, Francesco Bagnasco, Francesco Pera, Nicolò Di Tullio, Paolo Pesce

**Affiliations:** 1 Division of Implant Prosthodontics, Department of Surgical Sciences (DISC), University of Genoa, 16132 Genova, Italy; maria.menini@unige.it (M.M.); fcbagnat@gmail.com (F.B.); paolo.pesce@unige.it (P.P.); 2 Interdepartmental Research Center, Dental-School, University of Turin, 10126 Turin, Italy; francesco.pera@unito.it; 3 Department of Health Sciences (DISSAL), University of Genoa, 16132 Genoa, Italy; nicolo.ditullio@gmail.com

**Keywords:** microbiota, peri-implant, dental implants, full-arch, prosthesis, peri-implantitis

## Abstract

Background: The etiology of peri-implantitis is multifactorial, and it is not directly linked to the quantitative amount of plaque. The aim of this study was to evaluate the influence of subgingival microbiota around implants supporting full-arch restorations on clinical indexes of peri-implant health. Method: 47 patients (54 full-arch fixed rehabilitations) were included. Based on the highest value of probing depth (PD), 47 implants (in the test arch), 40 natural teeth and 7 implants (in the antagonist arch) were selected for microbiological sampling (traditional PCR and real-time PCR). Periodontal indexes (plaque index, PlI; probing depth, PD; bleeding on probing, BOP; peri-implant suppuration, PS) and marginal bone loss were also recorded. Results: Despite abundant plaque accumulation, the peri-implant parameters were within normal limits. No statistical difference was found in the microbial population around the test implants and antagonist natural teeth. Treponema denticola was present in a significantly higher amount around implants with increased PlI. Implants with increased BOP showed a significant increase in Treponema denticola and Tannerella forsythia. A significantly higher presence of Porphyromonas gingivalis, Treponema denticola and Tannerella forsythia was identified around the implants affected by peri-implantitis and in smokers. Conclusions: Peri-implantitis is characterized by a complex and polymicrobial disease, that might be influenced by the qualitative profile of plaque. Smoking might also favor implant biological complications in full-arch fixed prosthesis.

## 1. Introduction

For many years, dental implants have been a predictable therapeutic option for the fixed rehabilitation of patients with partial or complete edentulous jaws, and one of the mainstays of long-term dental implant success is the health of soft and hard tissues around osseointegrated implants [1].

Peri-implant tissue health can be threatened by two feasible pathologic conditions: mucositis and peri-implantitis, the latter being characterized by the progressive lack of supporting bone [2].

However, the clinical definition of the term peri-implantitis and the prevalence of peri-implantitis remain controversial, due to the lack of univocal criteria of diagnosis. Furthermore, the etiology of this process is still debated by the scientific community, and today it [3] should be considered multifactorial, with some risk factors common to periodontitis, such as poor oral hygiene, smoking, diabetes, and individual predisposition [4]. Some other contributing factors are specific for implants, like surgical trauma, mal-positioning of the implant, biomechanical factors, cement remnants, implant characteristics or the absence of a proper biological seal around implants [2,5].

In the past, several studies demonstrated a correlation between pathogenic bacteria and the development of peri-implant disease, but they considered the plaque control as the only cornerstone to prevent complications and implant failure [2]. However, according to recent literature, even if mucositis can be related to plaque accumulation, the amount of plaque is not directly correlated with peri-implantitis and plaque accumulation alone does not cause peri-implant bone resorption. A study conducted by Menini et al. showed successful clinical outcomes in patients treated with full-arch immediately loaded rehabilitations, recording no augmented bone resorption despite the presence of high levels of plaque around implants [6]. In addition, even if a proper oral hygiene is important, it is not always easy to maintain over time, especially in the case of old patients treated with fixed implant-supported full-arch prosthesis. 

A recent epigenetic study [7] showed how some specific miRNA profiles, sourced from peri-implant tissues, were associated with normal bone levels despite high plaque accumulation, underlying the importance of individual characteristics in affecting dental implants outcomes.

Another recent 10 year follow-up report of full-arch immediate loading rehabilitations of the upper jaw revealed that bone loss mainly occurred during the first months after implant insertion and loading, underlying the importance of a good management of prosthodontic risk factors [8]. 

However, while it is well established that the quantitative level of plaque alone is not able to determine bone loss around implants, the influence of its qualitative characteristics on the onset and progression of peri-implant disease is still to be clarified.

Several studies investigated the microbiological characteristics of peri-implantitis, looking for similarities with periodontitis and healthy implants’ bacterial environment.

About 30 min after implant insertion, peri-implant tissue starts to be colonized by commensal microorganisms [9]. Microbiota around healthy implants is limited to a supramucosal level and is mainly composed of Gram-positive cocci and low rates of Gram-negative anaerobes (similar to that of healthy teeth) that can colonize peri-implant tissue from other sites of the oral cavity, through a mechanism of “bacterial translocation” [9,10]. 

Periodontopathic pathogens were found around implants affected by peri-implantitis, and were also more frequent in partially, as compared to fully edentulous patients with dental implants, suggesting that periodontal pockets can replenish the peri-implant tissues of such species [11].

In both peri-implantitis and periodontitis there is a predominance of Gram-negative and anaerobic bacteria [12]. However, these similarities are no longer considered a crucial factor in the onset of peri-implant pathology. In fact, even if putative bacteria responsible for periodontitis [13] are frequently present in peri-implantitis in the early stage of disease, the core microbiota of the two conditions in the more advanced phases are clearly distinct [4]. For example, despite a co-occurrence by red complex microorganisms (such as *Porphyromonas gingivalis*, *Treponema denticola*, and *Tannerella forsythia*), a stronger association with orange complex members (such as *Prevotella intermedia* and *Fusobacterium nucleatum*) has been shown in peri-implant lesions [12].

In a recent study based on the 16S rDNA gene sequence, even if some microorganisms were recognized in both diseases, some of them dominated in peri-implantitis (*Actinomyces massiliensis*, *Porphyromonas* sp. *HOT-395*, *Prevotella nigrescens* and *Prevotella oris*) [14]. 

Other studies recovered a higher frequency of opportunistic microorganisms, such as enteric rods, *Pseudomonas aeruginosa*, *Staphylococcus aureus* and *Candida albicans* in peri-implantitis samples compared with periodontitis samples [12,15].

A recent systematic review identified the presence of Gram-negative bacteria, like *Aggregatibacter actinomycetemcomitans*, *Parvimonas micra*, and *Campylobacter rectus* in diseased peri-implant sites, in more than half of the considered studies [12]. 

Another systematic review has also found a frequent association of periodontitis with the Epstein–Barr virus and non-saccharolytic anaerobic Gram-positive rods [16].

After all, it can be asserted that peri-implantitis is a heterogeneous infection characterized by a different and more complex set of microorganisms, mainly non-cultivable Gram-negative bacteria, compared to periodontitis. 

However, nowadays a specific and comprehensive microbiological profile for peri-implant disease remains to be identified. Inter-individual differences in oral microorganisms, the oral environment complexity, the diversity of methods of sample analysis, also with the presence of non-cultivable or unknown species colonizing peri-implants lesions, render it extremely difficult. In addition, despite the eventual presence of a few microorganisms specific to peri-implant disease, a more complex pathogenic mechanism of polymicrobial synergy and dysbiosis has been recently suggested [17]. 

It has been proven that transition to pathology is related to bacterial endotoxins and host response mechanisms in peri-implant sites [10]. Therefore, in an equal bacterial component, an aberrant host response may contribute to the destruction of peri-implant tissue [18].

Clarifying the microbial ecology associated to peri-implantitis and its development could be essential for its prevention and treatment. 

A microbiological test from the peri-implant sulcus could be used as a rapid, non-invasive method for the diagnosis and risk assessment in the early stages of disease [19]. 

The primary aim of the present study was to evaluate the qualitative characteristics of plaque around implants supporting full-arch fixed prosthesis, investigating the possible correlations between different bacteria and the clinical indexes of peri-implant tissue health.

The secondary aim was to find a correlation between the microbiological pattern around dental implants and a smoking habit.

## 2. Methods and Materials 

### 2.1. Study Design and Population 

The present study was conducted between September 2018 and June 2019 at the Division of Implant and Prosthetic Dentistry, Department of Surgical Sciences (DISC) of Genoa University, Italy. 

**Figure 1:**
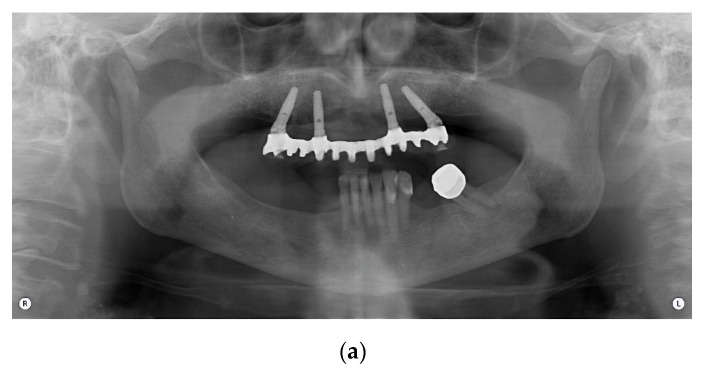

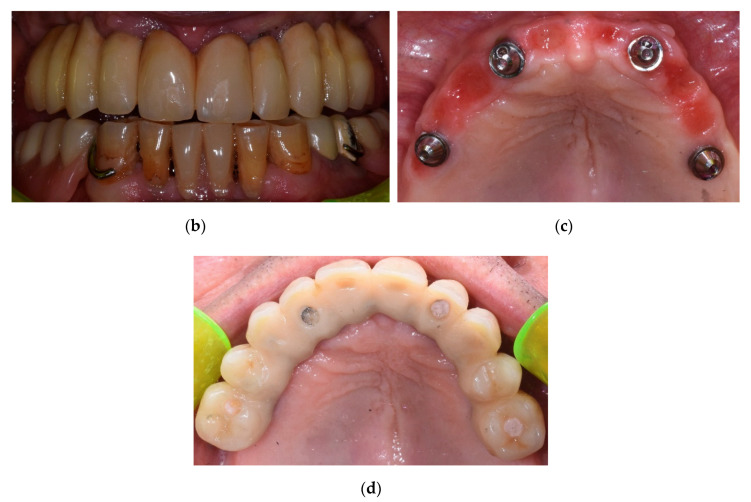
Panoramic radiograph of a full-arch immediate loading rehabilitation of the upper jaw according to the Columbus Bridge Protocol (**a**); frontal view (**b**) and occlusal intraoral views of the patient with (**d**) and without (**c**) prosthesis.

This study included 47 patients treated with an implant-supported prosthetic rehabilitation, according to the Columbus Bridge Protocol (CBP) [8,20] in the lower or upper jaw or in both dental arches (Figure 1).

The CBP protocol provides an immediately loaded full-arch, screw-retained prosthesis in the atrophic maxilla or mandible, supported by a reduced number of implants (4 to 6), with an expected delivery 24 h after surgery. Distal implants are mesiodistally tilted in order to insert long implants with high primary stability in native bone. Bone-grafting procedures are avoided. The screw-retained prosthesis is provided with a metal framework and a composite resin veneering material. 

At the time of the discharge, the patients received detailed guidance about hygiene and dietetic measures for the proper healing and maintenance of the rehabilitation and the long-term health of peri-implant tissues [21].

During the recall for periodic professional oral hygiene, eligible patients were asked to be enrolled in the present research. The inclusion criteria were the following:

1. ≥18 years of age;
2. Good general health, with ASA (American Society of Anesthesiologists) level of risk < 2;
3. Patients rehabilitated with an implant-supported full-arch prosthesis according to the Columbus Bridge Protocol (CBP) in the maxilla and/or mandible at least 12 months previously;
4. Patients who have been rehabilitated with implant-supported full-arch prostheses according to the Columbus Bridge Protocol (CBP) in the maxilla and/or mandible since at least 4 months;
5. Presence of peri-apical radiographs taken at the time of implant insertion or at the time of prosthesis delivery (24 h after implant insertion) (T0).

The exclusion criteria were the following:

- Patients affected by viral hepatitis B and/or C, HIV, coagulation problems, cardiovascular and/or systemic autoimmune diseases with or without oral tissue involvement (e.g., systemic lupus erythematosus, lichen ruber planus, HIV);
- Patients who have been diagnosed with myocardial infarction and/or cancer within the last 12 months;
- Biologic complications (e.g., failure of osseointegration process, implant mobility or loss) affecting one or more implants after the delivery of the implant-supported prostheses;
- Pregnant or in lactation patients;
- Antibiotic therapy during the last 5 days.

Smokers (<20 cigarettes/day) were not excluded from the research, however, patients were strongly encouraged to refrain from smoking. Heavy smokers (≥20 cigarettes/day) were excluded.

The present research was conducted in full agreement with the World Medical Association Declaration of Helsinki and received the approval of the local ethical committee.

Patients were widely and clearly instructed on the clinical procedure, and signed an understanding consent form before the enrollment in the experimental protocol.

No additional charges were applied for being included in the present research.

Peri-implant health parameters were recorded and microbiological samples were taken before professional oral hygiene.

### 2.2. Periodontal Parameters Recording

The screw-retained full-arch prostheses were removed from the mandible and/or maxilla, in order to record peri-implant health parameters. 

Probing depth (PD) was recorded with a plastic probe (Perio-Probe plastix 213718, Kerr Dental, Orange, CA, USA) at 4 sites (mesial, distal, lingual, buccal), on each implant/abutment, applying a force of about 0.2 N.

Plaque index (PlI) was evaluated through the use of a disclosing solution (Butler GUM Red-Cote liquid, Sunstar Americas Inc., Schaumburg, IL, USA) applied with a cotton bud, at the same 4 sites for each implant/abutment. Peri-implant bleeding on probing (BOP) was also evaluated at the same 4 sites for each implant/abutment. For both PlI and BOP, values from 0 to 4 were recorded, depending on the number of implant surfaces presenting plaque/bleeding, respectively.

Peri-implant tissue-suppuration (PS) was dichotomously assessed (present/absent). 

PlI was assessed after the microbiological sample collection (see below), in order to avoid the field contamination by the disclosing solution.

The type of prosthesis (i.e., “Natural Bridge” prosthesis, simulating teeth only, or “Toronto Bridge” prosthesis, replacing both teeth and soft tissues, thanks to a pink resin part) was also recorded.

At this time point, the clinicians also noted if the full-arch prosthesis was placed in the upper and/or inferior jaw, and if eventual biological and mechanical complications were present. In particular, possible mobility of the implants or abutment screws loosening were assessed.

PD was also measured around each natural tooth/implant of the antagonist arch. IP, BOP and PS were then recorded at the tooth with the highest PD score, following the same procedures described above for the implant sites. If the patient presented only dental implants and no natural teeth in the antagonist arch, periodontal parameters were recorded at the implant with the greater PD value.

Intraoral radiographs were taken, in order to compare the peri-implant bone level at the time of the present investigation (T1) with that measured on radiographs taken at T0 (that is the time of implant insertion or 24 h later at the time of the fixed prosthesis delivery). 

Interproximal bone level was recorded measuring the distance between a reference point (the implant–abutment interface) and the most coronal bone at the mesial and distal aspect of each implant. Bone resorption was measured as the difference between T0 and T1 bone level. For more details about bone resorption measurement, see a further article on immediately loaded full-arch rehabilitations previously published by the authors [22].

For all continuous variables recorded, the authors arbitrarily established cut-off values in order to turn them into categorical ones as described in a previous paper (Menini et al., 2019) [7]:

- Mean probing depth (PD): normal/increased (normal ≤ 3 mm, increased > 3 mm);
- Plaque index (PlI): low/high (low ≤ 1, high > 1);
- Bleeding on probing (BOP): present/absent (present > 0.0, absent = 0.0);
- Mean bone resorption (BR): normal/increased (normal ≤ 1 mm, increased > 1 mm);

Based on this, alternative categories (i.e., implants with normal PD versus implants with increased PD) were determined.

In addition, the authors considered all implants with the contemporary presence of BOP, altered PD and increased bone resorption as affected by peri-implantitis (PITI).

### 2.3. Microbiological Samples Collection

The instructions provided to the patients in view of the microbiological sample collection were the following:

- Do not eat, drink, chew gum and do not rinse the mouth with any mouthwash for at least 1 h before the sample is taken;
- Do not take antibiotics for at least 5 days before the sample is taken. In case the patient could not avoid antibiotic therapy, the patient was excluded from the study.

For each patient, only the implant and the natural tooth in the opposite arch which had the highest probing depth (PD) were selected for the microbiological sample. If the patient had only dental implants and no natural teeth in the antagonist arch, the microbiological samples were taken at the implant site with the greater PD value.

The microbiological sample collection was carried out according to the following procedures:

- Isolate the selected implant/tooth by aspiration and the use of cotton rolls, in order to avoid saliva contamination. Gently dry using an air syringe for at least 10 s;
- Insert 4 sterile endodontic paper points (ProTaper Gold Paper Points F1—Dentsply Sirona Italia Srl) in the peri-implant/gingival sulcus of each tooth/implant, placing them at the mesial, distal, lingual/palatal and buccal sites;
- After 30 s, remove the 4 paper points with a tweezer and put them in a sterile plastic tube and seal it immediately;
- Repeat the same procedure for the selected tooth/implant in the antagonist arch.

Once the operation was completed, 2 test tubes for each patient (each containing 4 paper points) were sent for microbiological analysis. 

### 2.4. Microbiological Analysis

The samples were sent to the Microbiology Laboratory of the Department of Surgical Sciences (DISC) of Genoa University, in order to analyze for the presence of putative bacteria responsible for peri-implant disease.

Paper points presenting blood contamination were excluded.

Two paper points for each sample were put in an Eppendorf tube containing 200 µL of Lysis Buffer at a temperature of 95 °C for 15 min, in order to cause the rupture of bacterial cells and extract their DNA. 

DNA was then isolated by centrifugation and analyzed by PCR (polymerase chain reaction). This biomolecular technique is based on an enzymatic reaction, in which a heat-resistant DNA polymerase (Taq Polymerase) catalyzes the specific amplification of known DNA sequences.

The PCR reaction occurred in a thermocycler containing a mix of the following components for each sample:

- A minimal amount of DNA being amplified;
- A buffer, in order to keep the pH stable and create the ideal conditions for the reaction;
- Elements such as magnesium ions (Mg^2+^), for the proper functionality of the polymerase;
- Nucleotides for the new DNA strands polymerization;
- Initiating primers;
- A Taq polymerase;
- A specific complementary DNA probe (for quantitative real-time PCR only, see below).

In the thermocycler, a varying number of cycles, consisting in the following 3 phases, were repeated:

1. Double DNA strand denaturation (at 95 °C);
2. Annealing of complementary primers to the 5′ and 3′ ends in the DNA sequence being amplified (at a varying temperature of 50–60 °C, depending on the type of primer);
3. DNA elongation by the enzyme Taq Polymerase.

Two different types of PCR were carried out in the present research:

(1) Traditional PCR: this analysis was made in order to detect the DNA of green and orange complex bacteria in addition to some opportunistic species: *Eikenella corrodens* (EC); *Campylobacter rectus* (CR); *Parvimonas micra* (PM); *Prevotella nigrescens* (PN); *Streptococcus mutans* (SM); *Staphylococcus epidermidis* (SE); *Stafilococcus aureus* (SA); *Fusobacterium nucleatum* (FN); and *Candida albicans* (CA). These species were called “Group 1”. Traditional PCR is a qualitative technique with the capability of assessing the presence or absence of each microbial species in the sample.

(2) Quantitative real-time PCR: This analysis was made in order to detect and simultaneously quantify the DNA of the following red and orange bacteria: *Aggregatibacter actinomycetemcomitans* (AA); *Prevotella intermedia* (PI); *Tanarella forsythia* (TF); *Treponema denticola* (TD); and *Porphyromonas gingivalis* (PG). These species were called “*Group 2*”. Real-time PCR is a more specific and sensible technique, as it reduces the time required for analysis compared with traditional PCR and it evaluates both the eventual presence and the quantity of bacteria. 

The traditional PCR amplicons (a DNA sequence can be amplified more than 100 times) were finally analyzed in agarose gel electrophoresis, while real-time PCR results were obtained by a digital software which provides the graphic reading of the fluorescent signal emitted by the fluorophores linked to the complementary DNA probe.

### 2.5. Statistical Methods

The descriptive statistical analysis included age, gender, smoking habit, implant or teeth position, presence/absence of an implant abutment and type of prosthesis (i.e., “Natural Bridge” versus “Toronto Bridge”). Peri-implant health parameters such as BOP, PD, PlI, PS and BR were analyzed, as well as the characteristics of the microbial population at the selected implant and dental sites.

Mean with the standard deviation or median with the interquartile range (IQR; 25–75th percentile) were reported for quantitative characteristics.

Student’s t test, Mann–Whitney (U) (for continues variables) and chi-square (for dichotomous variables) were also performed, in order to investigate the microbial correlation to peri-implant parameters and smoking habit. A generalized estimating equation (GEE) was finally used in order to investigate intrasubject interactions.

*p* < 0.05 was considered statistically significant and SPSS Statistics (Statistical Package for Social Science, v.21, IBM) was used for the computation.

## 3. Results

### 3.1. Baseline Population Characteristics

A consecutive cohort of 47 patients (26 males and 21 females; mean age: 64.34 years; SD: 9.61; range: 45–86 years), with a total of 218 implants, fulfilled the inclusion criteria and accepted to be enrolled in the present research. Five patients fulfilling the inclusion and exclusion criteria did not accept to participate in the study as they were not willing to remove their fixed prosthesis. 

Seven patients presented a CBP prostheses in both the upper and lower arch. Therefore, 54 CBP prostheses were evaluated in the study. Each CBP prosthesis was supported by four (95.7%) or five (4.3%) implants, and 96% (n = 45) of them were in the maxilla.

The mean time elapsed since the CBP implants’ insertion was 4.31 years (range: 1–15 years).

The antagonist arch was represented by:

- Natural teeth only (53%, n = 25 patients); in nine patients the natural teeth supported a partial removable prosthesis;
- Natural teeth and partial fixed prosthesis supported by natural teeth or implants (32%, n = 15);
- CBP prosthesis (15%, n = 7).

The seven patients with CBP prostheses in both dental arches were named the “double CBP” (DCBP) group and did not have any residual natural tooth. All the other 40 patients were considered the “CBP group”.

All the implants were stable and in function and no technical nor biological complications were encountered.

Based on the highest value of probing depth (PD), 47 implants were selected in the CBP arch, and 40 natural teeth (in the CBP group) and 7 implants (in the DCBP group) were selected in the antagonist arch for microbiological sampling, for a total of 94 microbiological samples. Therefore, in total, 14 implants were analyzed in the DCBP group (one in each dental arch), and 40 implants and 40 antagonist teeth were analyzed in the CBP group.

All the implants had a length ≥ 11.5 mm, were made of titanium and presented a modified surface. No machined implants were present. 

The main characteristics of the sample population are summarized in Table 1.

### 3.2. Assessment of Microorganisms around Implants and Natural Teeth

All the patients followed the provided instructions in view of the microbiological sampling. Results of the microbiological analysis are reported in Table 2.

According to the Mann–Whitney U test and the chi-square test, no statistical difference was found in the bacterial population around CBP implants and antagonist natural teeth.

A generalized estimating equation (GEE) was then used, in order to find an intrasubject difference in the microbial species and bacteria among CBP implants versus natural teeth. However, no statistical difference emerged, neither for “Group 1” (*p* = 0.388) microbes nor for “Group 2” bacteria (*p* = 0.651).

No statistical difference was found even in the microbial population around the implants of the CBP group and implants of the DCBP group. For this calculation, for each patient of the DCBP group, only the implant with the highest PD value was considered.

### 3.3. Microbial Correlation to Peri-Implant Parameters

Mean values of the peri-implant parameters recorded for the 40 implants with the highest PD value in the CBP group (n = 40) are reported in Table 3. Despite the relevant rate of plaque accumulation (PI), the parameters of peri-implant soft tissue health (PD, BOP and PS), as well as BR were within the normal limits at the majority of the implant sites (Table 3). 

The peri-implant parameters recorded for the CBP implants (n = 40) were dichotomized as described in the Materials and Methods Section, in order to investigate a correlation between the microbiological results and BR, PD, PI and BOP. The Mann–Whitney U test and chi-square test did not show any significant difference in the microbial pattern between the implants with an increased or normal PD and BR.

Concerning PI, TD was the only bacteria present in a significantly higher amount around implants with an increased PI (*p* = 0.004).

Implants with an increased BOP showed a significantly increased presence of TD (*p* = 0.001), TF (*p* = 0.042), and the totality of “Group 2” microorganisms (*p* = 0.023) (Table 4).

### 3.4. Differences in Microbial Biofilms of Implants with or without Peri-Implantitis (PITI)

The difference in the microbial pattern around CBP implants (n = 40) affected or not affected by PITI was investigated. According to the cut-off values applied in the present study, PITI was detected in five implants (12.5%). However, it must be noted that considering the criteria proposed during the 2017 World Workshop on the classification of periodontal and peri-implant diseases and conditions (Berglundh et al., 2018) [23], none of the implants included in the present study would have been considered affected by peri-implantitis, as further discussed in the Discussion Section. 

Regarding “Group 1” microbes, no statistically significant differences were detected between the CBP implants with and without PITI.

Concerning the “Group 2” bacterial species, implants affected by PITI showed a significantly higher presence of PG, TF, TD and the totality of “Group 2” microorganisms (Table 5 and Table 6). All these bacteria were on average present in a reduced amount around implants free of disease.

### 3.5. Correlation between Biofilm around Implants and Smoking Habit

A further investigation was made, in order to assess if smokers showed a different bacterial population around the selected implants (n = 54) when compared to non-smokers, as smoking is considered one of the possible risk factors in promoting peri-implant disease [24].

Mann–Whitney U test showed a significant difference between smokers and non-smokers for microbial tests for PG, TF, TD, and the totality of the “Group 2” microorganisms (Table 7). While TF was more present in non-smoking patients, all other cited bacteria were significantly more present in smokers.

The chi-square test showed a non-significant difference for the presence/absence of “Group 1” microorganisms (CR, EC, FN, PM). For SA, SM, SE, CA, the test was not applicable due to data distribution.

## 4. Discussion

In the present study, a consecutive cohort of 47 patients rehabilitated with implant-supported full-arch prostheses were recruited and underwent a microbiological analysis at the implant and dental sites with higher PD.

The primary aim of this study was to investigate the possible correlations between the bacterial profile of biofilm around dental implants and peri-implant health parameters (PlI, BOP, PD and bone resorption), in order to identify the eventual microbiological risk factors for the development of peri-implant disease. It must be underlined that in each dental arch, microorganisms were harvested only from the implant/dental site with the highest PD values. This may have affected the outcomes.

In a systematic review concerning microbial biofilm profiles around dental implants affected by peri-implantitis, the major periodontopathic microorganisms of red complex (TF), (TD) and (PG), orange complex (PI, (FN)), and other microorganisms (PM, CR, (EC) and AA) were identified in more than half of the included studies [12].

All the cited microorganisms were also analyzed and identified in the present research and in particular, PN and TF were the two most present species, both around CBP implants and in antagonist natural teeth. On the contrary, SM, SE, CA, SA were never detected, in any sample taken, even if some previous studies had shown the presence of these microorganisms in peri-implant sites [12].

In addition, no statistical differences were found between the bacterial species around CBP implants and DCBP implants or natural teeth, not even at an intra-individual level. This is consistent with previous studies, which have demonstrated similarities between the microbial pattern around teeth and implants, suggesting that a bacterial transmigration process from tooth to implants is possible [25]. However, Apatzidou et al. recently showed that diseased implants have distinct microbiological ecosystems from healthy periodontal tissues in people with previous periodontitis [26].

On the other hand, some recent studies have proven that both healthy implants and peri-implantitis are colonized by periodontopathic microorganisms, and as a consequence it also seems that well osseointegrated implants with healthy peri-implant tissues can be colonized by microbial species, that are typically responsible for periodontal disease [12,27].

The present study is partly in agreement with these findings, in fact the presence of microorganisms related to periodontitis was detected in both healthy CBP implants and those affected by PITI (12.5%, n = 5). However, a significant difference was shown for “Group 2” bacteria (*p* = 0.004), which included the major periodontopathic microorganisms of the red and orange complex that were significantly more present in the implant sites affected by PITI. In particular, an increase in the frequency was observed for PG (*p* = 0.013), TD (*p* = <0.001) and TF (*p* = 0.002), which constituted the 40.5% of the peri-implant microbiota.

Other studies described the peculiar presence of these three bacteria in infected peri-implant sites, when compared to healthy peri-implant tissues, and one study also showed a virulence cooperation between them, resulting in immunoinflammatory bone resorption [28].

Comparing healthy implants and the implants affected by PITI, no statistical difference was found for “Group 1” bacteria. This is in contrast with the paper of Canullo et al., who found a higher rate of PM, PI, and FN in peri-implantitis, compared with healthy implants biofilm [29]. In our study, only the presence of PN, a less virulent member of orange complex, was near to statistical significance in PITI sites (*p* = 0.053), supporting the findings of a previous study which also showed an increased abundance of PN in PITI than periodontitis [30].

Costa et al., in a recent 5 year follow-up clinical trial, showed high frequencies of PG, TD and FN in people who progressed from peri-implant mucositis to peri-implantitis and highlighted a beneficial role of preventive maintenance therapy. These microbial species were also correlated to probing pocket depth, bleeding on probing, and radiographic bone loss [31].

The present research, on the contrary, found a microbial correlation only with PlI and BOP.

The levels of plaque accumulation assessed were high (mean PlI: 2.1 ± 1.5; 52.5%), but TD was the only more abundant bacteria around implants with an increased PlI (*p* = 0.004).

In addition, the present study found a significant correlation between the BOP and an increase in the frequencies of TD (*p* = 0.001), TF (*p* = 0.042), and the totality of “Group 2” microorganisms (*p* = 0.023).

Despite the relevant rate of plaque accumulation, the parameters of peri-implant soft tissue disease, PD (mean 2.47 ± 0.73 mm) and BOP (mean 0.39 ± 0.84; 9.7%), were within normal limits for the majority of the implant sites. The majority of the implants was not affected by PITI and showed normal peri-implant bone levels (mean BR = 1.240 ± 0.87 mm).

This is in agreement with the previous findings of the authors, in which high levels of plaque amount were not related to peri-implant bone resorption [6].

Despite this, it is interesting to note that the two major bacteria related to plaque accumulation (TD) and BOP (TD and TF) were also present in high frequencies in implants affected by PITI. As a consequence, despite that the cross-sectional methodological design of the present study is not able to establish a cause–effect relationship, it is reasonable to think that the plaque qualitative profile might be influential in the development of PITI. Peri-implantitis is an intra-individual and polymicrobial infection, in which a wide range of factors, in addition to the subjective host response, concur in promoting the onset of the disease. In particular, specific individual factors have been demonstrated to determine a predisposition or a resistance towards peri-implantitis [7].

Good professional and home oral hygiene may be essential in providing the clinical and ecological stability of peri-implant tissues over time, however, it is not easy to maintain a proper domiciliary oral hygiene in the case of full-arch fixed prosthesis supported by dental implants (especially for older patients), and clear protocols of home and professional hygienic management in this kind of rehabilitation are still to be defined.

Regarding the common disagreement on the diagnosis and definition of peri-implantitis, it must be underlined that in the present study, we considered affected by peri-implantitis (PITI) all implants with the contemporary presence of BOP, increased PD and increased bone resorption, based on the arbitrary cut-off values reported in the Materials and Methods Section. The choice of such cut-off values was made in accordance with previous studies published by the same team of authors [7,32]. A direct correlation with the new classification proposed by the 2017 World Workshop on the classification of periodontal and peri-implant diseases and conditions [23] was not possible. In fact, in the present study, bone resorption was calculated comparing the peri-implant bone level at the time of the present investigation (T1) with that measured on radiographs taken at T0 (that is the time of implant insertion or 24 h later at the time of the immediate loading fixed prosthesis delivery). In contrast, Berglundh et al. [23] recommend that “the clinician obtain baseline radiographic and probing measurements following the completion of the implant-supported prosthesis” referring to delayed loading prostheses, that is after the initial remodeling phase.

Berglundh et al. [23] additionally proposed a definition to be used in the absence of previous examination data. In this case, the new classification proposed by the 2017 World Workshop proposes stricter parameters to define peri-implantitis compared to the parameters used in the present research. In fact, in the present study we considered affected by PITI dental implants with PD > 3 mm and bone resorption > 1 mm. Berglundh et al. considered as affected by PITI dental implants with PD ≥ 6 mm and bone levels ≥ 3 mm apical of the most coronal portion of the intraosseous part of the implant. According to this latter definition, none of the implants included in the present study were affected by peri-implantitis, in fact the maximum value of PD recorded was 4.5 mm (see Table 3). It is interesting to underline that only the implants with the greatest PD value in their dental arch were included in the present evaluation, indicating that peri-implant tissue health was optimal in the present study.

The present study also investigated the influence of smoking on the peri-implant microbiological pattern and found a significant correlation of smoking habit with PG (*p* = 0.001), TD (*p* = 0.006), and the totality of “Group 2” microorganisms (*p* = 0.010). On the contrary, TF was more present in non-smoking patients (*p* = 0.028).

This is consistent with several studies, reporting that smoking negatively affects the subgingival microbiome in both healthy and diseased peri-implant sites, depleting the commensal species and supporting a pathogen-rich community [33,34,35].

According to Balshe et al., smokers have an increased risk of failure of implants with a machined surface. Moreover, in the sample of 593 patients, implant survival was the poorest for implants placed in the maxillary posterior areas of smokers [35]. This may be due to multiple mechanisms induced by smoke, including a variation in the microbial population.

These findings confirm the need of appropriate measures to encourage patients to avoid smoking, but also suggest the eventual use of personalized therapy approaches in smokers and non-smoker patients.

Finally, microbial sampling from peri-implant sulcus may be a simple and non-invasive method to identify the presence of microbial profiles associated with peri-implant disease, which could address more stringent preventive protocols and personalized anti-infectious treatments.

Further studies should investigate if different protocols for hygienic maintenance could modulate the microbiota around implants, supporting full-arch fixed prosthesis.

In addition, other newly proposed pathogenetic microbial species should be investigated, even if it is worth to specify that many of them are still to be cultivated, so they cannot be detected by molecular methods (PCR). Sequencing methods, such as 16S rRNA, have already been employed in order to overcome these limitations, identifying a wider range of microorganisms, including undetected and uncultivable bacteria (i.e., Bacteroidetes, Fusobacteria, Actinobacteria, Proteobacteria, Synergistetes, Spirochaetae, etc.) [36].

In the present research, putative less pathogenetic microorganisms were excluded from quantitative analysis by real-time PCR. This can be considered a limiting factor.

## 5. Conclusions

The outcomes of the present research highlighted that the major periodontopathic microorganisms of red complex, orange complex and other microorganisms, were present around both CBP implants and natural teeth, as well as in both diseased and healthy sites. However, PG, TD and TF were more related to PITI. High frequencies of TD were detected in the case of a high level of plaque accumulation, while the abundance of TD and TF was present around implants with increased BOP. PG and PD were significantly increased in smoking patients.

## Figures and Tables

**Table 1:** Baseline population characteristics.

**CBP Group (n: 40)**	**Mean**	**SD**	**Min**	**Max**	**%**
Age	64.34	9.61	45	86	
Gender (M)					55
Smokers					25
CBP Prosthesis (Natural Bridge)					95
Presence of Multi-Unit Abutment (MUA) *					95
**DCBP Group (n: 7)**	**Mean**	**SD**	**Min**	**Max**	**%**
Age	63.07	7.72	47	74	
Gender (M)					71
Smoker					57
CBP Prosthesis (Natural Bridge)					100
Presence of Multi-Unit Abutment (MUA)					100

* The two implant sites where the MUA was not present were rehabilitated directly on the implant head; Columbus Bridge Protocol (CBP) group: 40 patients with natural teeth in the antagonist arch; double CBP (DCBP) group: 7 patients with CBP prostheses in both dental arches; MUA: multi-unit abutment; Min: minimum value; Max: maximum value; SD: standard deviation.

**Table 2:** Assessment of the microorganisms around CBP implants and antagonist natural teeth and implants.

**Implants (n: 40)**	**Min–Max**	**Mean (SD)**	**% (+ Cases/40)**
CR			8 (3)
EC			5 (2)
FN			3 (1)
PM			0 (0)
PN			25 (10)
SA			0 (0)
SM			0 (0)
SE			0 (0)
CA			0 (0)
AA	0.00–49.000	2.550 (9.722)	2 (5)
PG	0.00–67,950.000	3002.900 (11,826.386)	7 (18)
PI	0.00–2770.000	108.450 (483.836)	3 (8)
TF	0.00–164,573.000	7001.180 (27,000.457)	15 (38)
TD	0.00–4642.000	267.200 (884.403)	11 (28)
“Group 2” tot.	0.00–40,946.200	2076.455 (7276.455)	17 (43)
**Antagonist Natural Teeth (n: 40)**	**Min–Max**	**Mean (SD)**	**% (+ Cases/40)**
CR			5 (2)
EC			15 (6)
FN			6 (2)
PM			3 (1)
PN			20 (8)
SA			0 (0)
SM			0 (0)
SE			0 (0)
CA			0 (0)
AA	0.00–69.000	3.630 (13.237)	2 (5)
PG	0.00–75,986.000	4800.630 (15,165.136)	10 (25)
PI	0.00–884.000	45.930 (155.593)	7 (18)
TF	0.00–590,869.000	19,680.560 (94,548.644)	22 (56)
TD	0.00–18,637.000	707.550 (3003.893)	10 (25)
“Group 2” tot.	0.00–123,228.600	4949.255 (19,674.790)	22 (55)
**Implants (n: 14)**	**Min–Max**	**Mean (SD)**	**% (Positive Cases/14)**
CR			0 (0)
EC			0 (0)
FN			0 (0)
PM			0 (0)
PN			1 (7)
SA			0 (0)
SM			0 (0)
SE			0 (0)
CA			0 (0)
AA	0.000–4.000	0.290 (1.069)	0 (0)
PG	0.000–24,942.000	2567.430 (7075.764)	2 (14)
PI	0.000–548.000	73.570 (155.740)	4 (29)
TF	0.000–5114.000	648.290 (1353.070)	7 (50)
TD	0.000–5735.000	588.570 (1530.708)	7 (50)
“Group 2” tot.	0.000–5838.200	775.629 (1561.634)	7 (50)

Mean (SD) and the percentage of positive cases. A microbiological sample was considered as a “weak negative” when up to 19 bacterial copies/μL were detected, while “weak positive” when the copies/μL were above this threshold; Min: minimum value; Max: maximum value; SD: standard deviation.

**Table 3:** Peri-implant health parameters (CBP implants, n = 40).

40 Implants	Min	Max	Mean	SD
BR (mm)	0.00	3.01	1.240	0.87
PlI	0	4	2.1 (52.5%)	1.5
PS	0.0	0.5	0.01	0.05
BOP	0.0	4.0	0.44 (9.5%)	0.84
PD (mm)	1.0	4.5	2.47	0.73

**Table 4:** Correlation between the microbial population and BOP in the CBP group. Percentage of positive cases (“Group 1” bacteria) and mean (SD) (“Group 2” bacteria) as evaluated in the 40 CBP implants.

Group 1 Microbes	Presence of BOP	Absence of BOP	Group 2 Bacteria	Presence of BOP	Absence of BOP
CR	7 (2)	11 (1)	AA	2 (9)	5 (13)
EC	3 (1)	11 (1)	PG	2462 (12,206)	4867 (10,866)
FN	3 (1)	0 (0)	PI	50 (248)	310 (923)
PM	0 (0)	0 (0)	TF	1690 (6645)	25,294 (53,876)
PN	23 (7)	33 (3)	TD	44 (157)	1036 (1692)
SA	0 (0)	0 (0)	“Group 2” tot.	849.600 (3740.928)	6302.289 (13,406.638)
SM	0 (0)	0 (0)			
SE	0 (0)	0 (0)			
CA	0 (0)	0 (0)			

**Table 5:** Differences in the microbial biofilms of implants with or without peri-implantitis (PITI) as evaluated in the CBP group (40 patients; 40 implants).

PITI versus Non PITI	X^2^ Test (*p* Value)	Mann–Whitney U (*p* Value)
CR	0.257	
EC	0.100	
FN	0.702	
PM	-	
PN	0.053	
SA	-	
SM	-	
SE	-	
CA	-	
AA		0.644
PG		0.013
PI		0.734
TF		0.002
TD		<0.001
“Group 2” tot.		0.004

**Table 6:** Qualitative and quantitative assessment of the microbial species in the implants with or without PITI in the CBP group (40 patients; 40 implants): percentage of positive cases (“Group 1”); mean (SD) (“Group 2”).

**Group 1 Microbes % (Positive Cases)**	**Non PITI**	**PITI**
CR	6 (2)	20 (1)
EC	3 (1)	20 (1)
FN	3 (1)	0 (0)
PM	0 (0)	0 (0)
PN	20 (7)	60 (3)
SA	0 (0)	0 (0)
SM	0 (0)	0 (0)
SE	0 (0)	0 (0)
CA	0 (0)	0 (0)
**Group 2 Microbes Mean (SD)**	**No PITI**	**PITI**
AA	3 (10)	1 (3)
PG	2180 (11,493)	8761 (13,811)
PI	45 (234)	554 (1239)
TF	2211 (7411)	40,529 (70,960)
TD	68 (219)	1661 (2108)
“Group 2” tot.	901.491 (3600.630)	10,301.200 (17,591.005)

**Table 7:** Correlation between microbial species and smoking habit. Mean (SD).

“Group 2” Bacteria	Non-Smokers	Smokers	*p* Value
AA	3.43 (12.46)	0.69 (2.04)	0.759
PG	2678.43 (11,092.10)	6382.77 (16,320.85)	0.001
PI	36.35 (176.21)	182.04 (565.26)	0.104
TF	11,544.96 (72,235.35)	10,890.50 (33,957.98)	0.028
TD	494.53 (2399.19)	523.15 (1117.16)	0.006
“Group 2” tot.	2917.58 (15,178.11)	3595.83 (8953.58)	0.010
